# Bacterial outer membrane vesicle biogenesis: a new mechanism and its implications

**DOI:** 10.15698/mic2016.06.508

**Published:** 2016-05-10

**Authors:** Sandro Roier, Franz G. Zingl, Fatih Cakar, Stefan Schild

**Affiliations:** 1 Institute of Molecular Biosciences, University of Graz, NAWI Graz, BioTechMed-Graz, Humboldtstraße 50, A-8010 Graz, Austria.

**Keywords:** outer membrane vesicles, OMV biogenesis, gram-negative bacteria, Haemophilus influenzae, phospholipids, host-pathogen interactions, vaccines

## Abstract

Outer membrane vesicle (OMV) release by Gram-negative bacteria has been observed and studied for decades. First considered as a by-product of cell lysis, it soon became evident that OMVs are actively secreted from the outer membrane (OM) of Gram-negative bacteria. Accordingly, these small particles (~ 10-300 nm in diameter) consist mainly of OM components like phospholipids (PLs), OM proteins, and lipopolysaccharides or lipooligosaccharides. However, OMVs may also comprise periplasmic, inner membrane, or cytoplasmic components. Since the shedding of substantial amounts of OM material represents a significant energy cost to the bacterial cell, OMV production must have some vital biological functions for Gram-negative bacteria. Indeed, intense research on that topic revealed that OMVs play important roles in bacterial physiology and pathogenesis, ranging from secretion and delivery of biomolecules (for example, toxins, DNA, or quorum sensing molecules) over stress response and biofilm formation to immunomodulation and adherence to host cells. Only recently researchers have begun to elucidate the mechanistic aspects of OMV formation, but a general mechanism for the biogenesis of these vesicles is still lacking. Here we review the findings and implications of our recent study published in Nature Communications (Roier S, *et al.* (2016) Nat. Commun. 7:10515), where we propose a novel and highly conserved bacterial OMV biogenesis mechanism based on PL accumulation in the outer leaflet of the OM. This mechanism might not only have important pathophysiological roles *in vivo*, but also represents the first general mechanism of OMV formation applicable to all Gram-negative bacteria.

Current OMV biogenesis models are based on either loss or relocation of covalent linkages between the OM and the underlying peptidoglycan layer, require an accumulation of peptidoglycan fragments or misfolded proteins in the periplasmic space, or need the enrichment of species-specific membrane curvature-inducing molecules. However, these models are limited by the fact that they either require genetic manipulations, the presence of stress, or are only applicable to a single bacterial species hitherto. In order to discover a general and conserved mechanism of OMV formation within Gram-negative bacteria, we screened transposon mutants of *Haemophilus influenzae*, an opportunistic pathogen of the human respiratory tract with a relatively small genome size of 1.83 Mb, for altered OMV production. Amongst others, we identified gene disruptions in *yrbB* and *yrbE* that seem to increase vesiculation. These two genes are part of a gene cluster comprising three additional genes (*yrbC*, *yrbD*, and *yrbF*). A previous study by Malinverni and Silhavy suggested that in *Escherichia coli*, homologs of these five gene products, together with the VacJ protein, are important for maintaining the lipid asymmetry in the Gram-negative OM. This highly conserved ABC (ATP-binding cassette) transport system is thought to prevent PL accumulation in the outer leaflet of the OM by retrograde trafficking of PLs from the OM to the inner membrane. Figure 1A illustrates the putative VacJ/Yrb ABC transport system in *H. influenzae* based on the published *E. coli* model.

**Figure 1:**
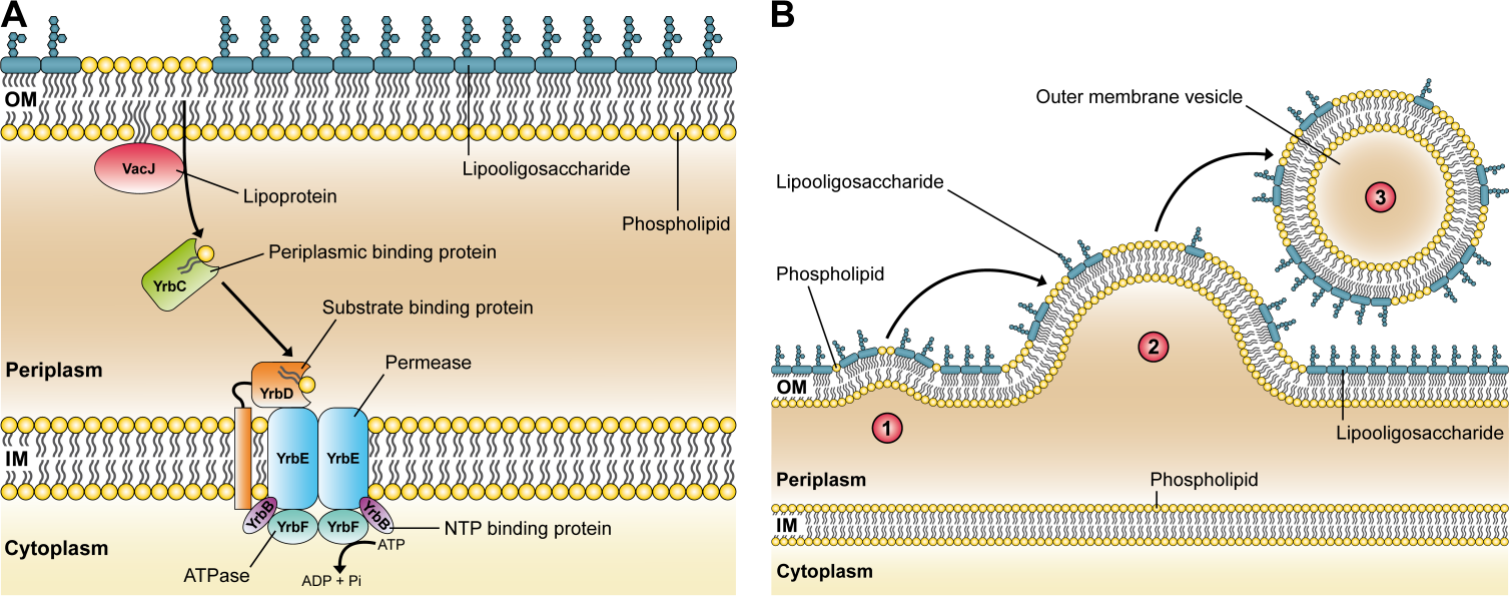
FIGURE 1: A conserved ABC transport system and its relevance for OMV biogenesis in Gram-negative bacteria. **(A)** Illustration of the VacJ/Yrb ABC transport system in *H. influenzae* that putatively prevents PL accumulation in the outer leaflet of the OM by retrograde trafficking of PLs from the OM to the inner membrane (IM). The model is based on findings in *E. coli* published by Malinverni and Silhavy. **(B)** A new model of OMV formation in Gram-negative bacteria. Step 1: Decreased expression or deletion of *vacJ* and/or *yrb* genes results in PL accumulation in the outer leaflet of the OM. This asymmetric expansion of the outer leaflet initiates an outward bulging of the OM. Step 2: Further enrichment of positive and negative curvature-inducing PLs in both leaflets supports the budding of the OM, which finally pinches off to form an OMV. Step 3: The released OMV is enriched in PLs incorporated into the outer leaflet of the vesicle membrane. Copyright disclaimer notice: the figure is composed of Figure 1a and Figure 8 of the original article (Roier *et. al*, doi:10.1038/ncomms10515), which is licensed under a Creative Commons Attribution 4.0 International License.

Sophisticated OMV quantification techniques in combination with gene-specific *vacJ* and *yrbE* deletion mutants in *H. influenzae* as well as in distantly related Gram-negative bacteria confirmed that disruptions within the VacJ/Yrb ABC transport system increase OMV production without compromising the OM integrity. Inversely, the overexpression of the *yrb* gene cluster in *E. coli* resulted in decreased vesiculation indicating a highly conserved key role of the VacJ/Yrb ABC transport system in OMV formation. Comparative OMV and OM composition analyses of the *H. influenzae* wild-type and PL transporter mutants ∆*vacJ* and ∆*yrbE* revealed that OMVs derived from the PL transporter mutants have an altered lipidome while the proteome does not vary significantly. In comparison with wild-type OMVs, we found that PL transporter mutant OMVs are similar sized but enriched in PLs, which implicates a PL incorporation into the outer leaflet of the vesicle membrane. In addition, we showed that certain fatty acids are enriched or reduced in PL transporter mutant OMVs, suggesting that defined PL rearrangements promote OMV formation in *H. influenzae*. All these findings allowed us to propose a novel OMV biogenesis mechanism in Gram-negative bacteria, which is based on an accumulation of PLs in the outer leaflet of the OM that promotes vesicle formation (Figure 1B). Since disruptions in the highly conserved VacJ/Yrb ABC transport system lead to similar hypervesiculation phenotypes in distantly related species, the proposed model could be the first general mechanism of OMV formation applicable to all Gram-negative bacteria.

Moreover, we demonstrated that this mechanism is regulated by iron availability in all Gram-negative bacteria tested. Our findings indicate that iron limitation leads to a ferric uptake regulator (Fur)-dependent downregulation of the VacJ/Yrb ABC transport system, which ultimately results in increased OMV production. In *H. influenzae*, we showed that the disadvantage of an increased serum sensitivity caused by this downregulation can be overcome *in vitro* by the addition of physiological concentrations of OMVs. Since our study also revealed that the expression levels of the PL transporter genes in *H. influenzae* are significantly decreased during the initial stages of nasopharyngeal colonization, we suggest that iron-limiting conditions in the host cause an increased OMV production by the bacteria *in vivo*. This hypervesiculation might have an important pathophysiological role during the transmission of *H. influenzae* into a new host. Increased OMV production could counteract antibody and complement attacks by binding and depleting these factors and thereby lowering the selective pressure of local immune defence mechanisms, which in turn facilitates colonization of the nasopharynx.

As iron limitation is a general stress faced by many microbial pathogens upon entry of the human host, it can be hypothesized that Gram-negative pathogens initiate vesiculation at early stages *in vivo*. This might explain, why several recent studies describe OMVs as delivery vehicles for virulence factors to host cells. Indeed, OMVs allow virulence factors to be transported in a concentrated and protected manner. It was shown that toxins can be more potent and biologically active when associated with vesicles than in their soluble form. For example, Shiga toxins of *Shigella dysenteriae* and enterohemorrhagic *E. coli* O157:H7 are protected from hydrolysis by host proteases when secreted by OMVs. Some virulence factors like the heat-labile enterotoxin (LT) of enterotoxigenic *E. coli* are preferably transported via OMVs. It is thought that LT-loaded OMVs trigger not only the internalization of the toxin, but also other bacterial vesicle components into the host cell. Complementary to our results, the expression of some OMV-associated virulence factors is influenced by iron availability. In *Helicobacter pylori*, it was demonstrated that the levels of the vacuolating cytotoxin A (VacA) are reduced, whereas two new proteolytic enzymes are expressed on these OMVs under iron-limiting conditions. These results indicate that environmental iron levels not only influence the quantity of OMVs, but can also change the composition of OMV-associated virulence factors.

Notably, OMV formation is not restricted to Gram-negative pathogens. It is tempting to speculate that iron limitation, or in a more general aspect nutrient limitation, was a driving evolutionary trigger for vesiculation. Already three decades ago, it was shown that starving bacteria release high amounts of OMVs, which was initially thought to enhance the fitness of the donor bacterium due to a reduction in size. Recently, it was found that the formation of OMVs during low nutrient conditions has additional functions for the survival of bacteria. For example, vesicles derived from *Mycobacterium tuberculosis*, *Neisseria meningitidis* and *Porphyromonas gingivalis* are associated with iron-scavenging proteins, whereas OMVs of *Pseudomonas fragi* contain proteases like xylanases or cellulases. Such predatory OMVs may provide a versatile mean for nutrient acquisition and therefore an important fitness advantage.

Besides its possible impact on bacterial physiology and pathogenesis, our proposed new mechanism for the biogenesis of OMVs in Gram-negative bacteria might also influence the development of new antibiotics or OMV-based vaccines in the future. Our group has already demonstrated that OMVs have a great potential to act as a vaccine against a broad range of bacterial infections. Now, with our new understanding of bacterial OMV formation, it is possible to increase the yield of OMVs without compromising their natural composition. Similarly, our findings might stimulate the development of novel antibacterial drugs targeting this pathophysiologically important OMV biogenesis mechanism.

